# Supplementary material for: Mitochondrial transplantation: adaptive bio-enhancement
Source: Cell Death Dis. 2025 Jul 1;16(1):473. doi: 10.1038/s41419-025-07643-8 (PMC12218056; doi:10.1038/s41419-025-07643-8)

Figure 3C

β-actin

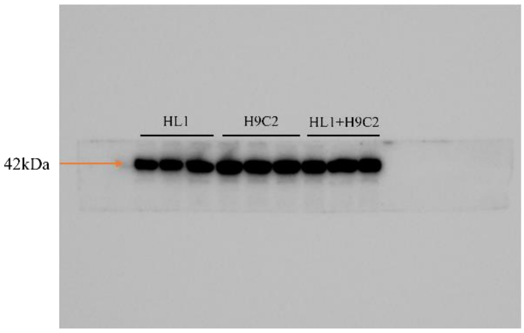

Drp1

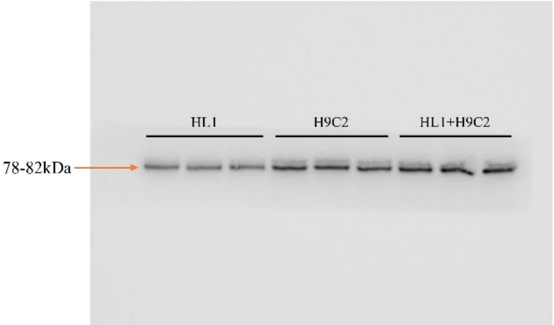

Figure 3C

Opa1

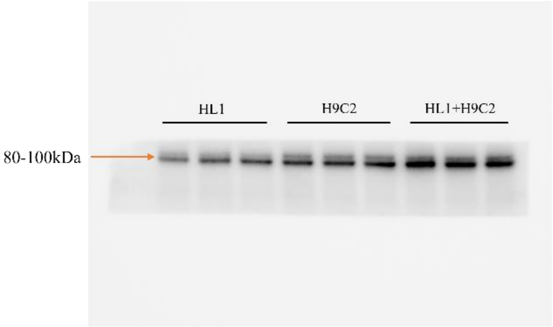

Mfn1

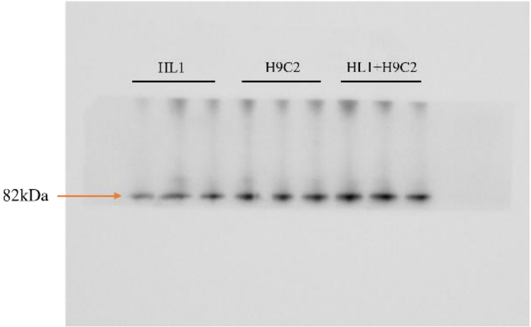

Figure 6I

LPS Model
Liver P-JNK

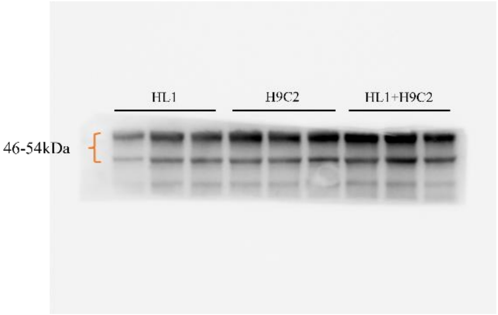

LPS Model
Liver JNK

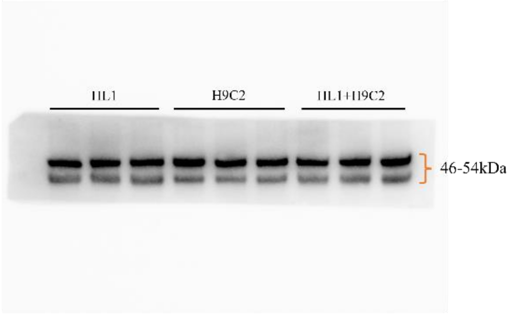

Figure 6I

LPS Model
Liver P-p65

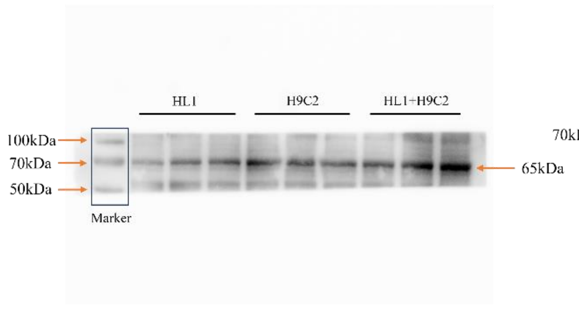

LPS Model
Liver p65

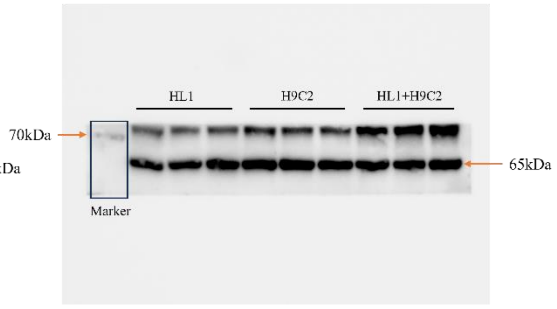

Figure 6I

LPS Model
Liver β-actin

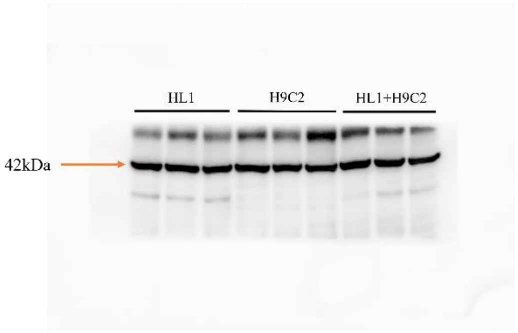

Normal Model
Liver β-actin

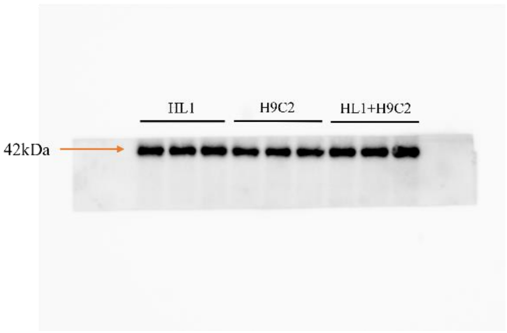

Figure 6I

Normal Model
Liver JNK

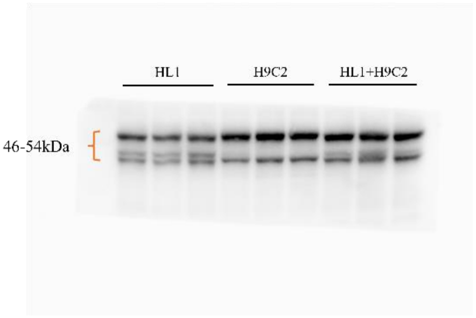

Normal Model
Liver P-JNK

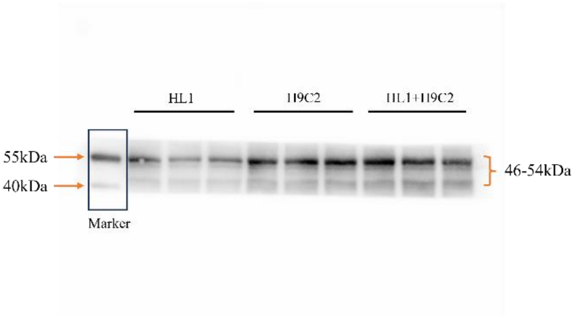

Figure 6I

Normal Model
Liver p65

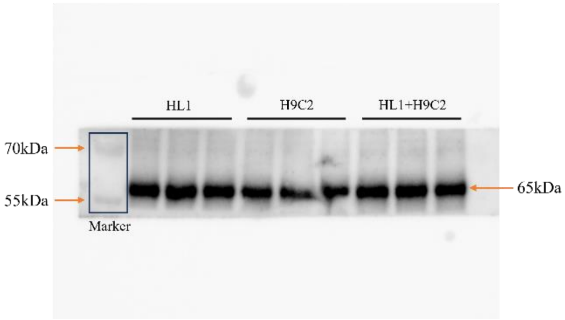

Normal Model
Liver P-p65

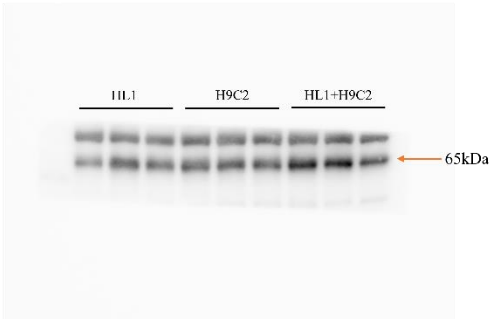

Figure 6I

Normal Model
Kidney P-JNK

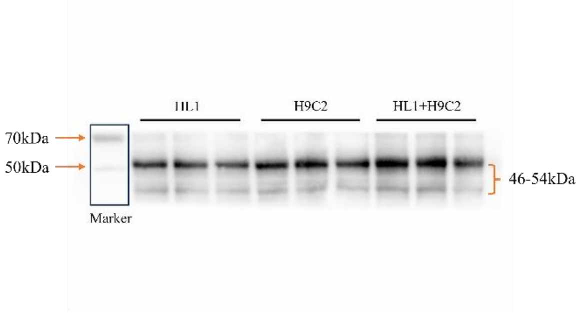

Normal Model
Kidney JNK

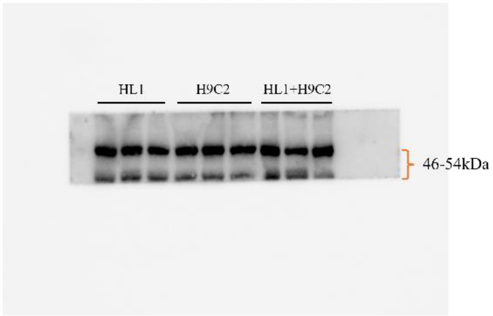

Figure 6I

Normal Model
Kidney P-p65

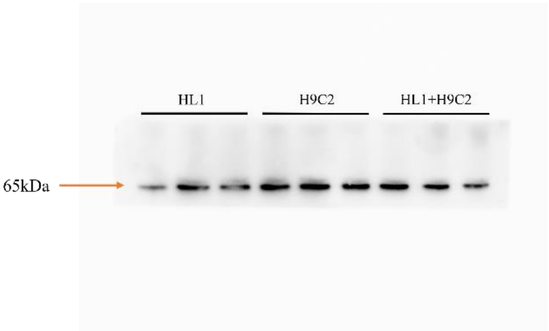

Normal Model
Kidney p65

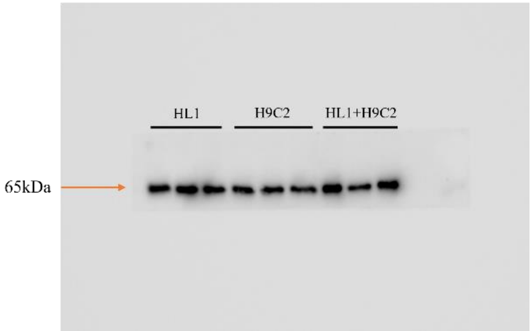

Figure 6I

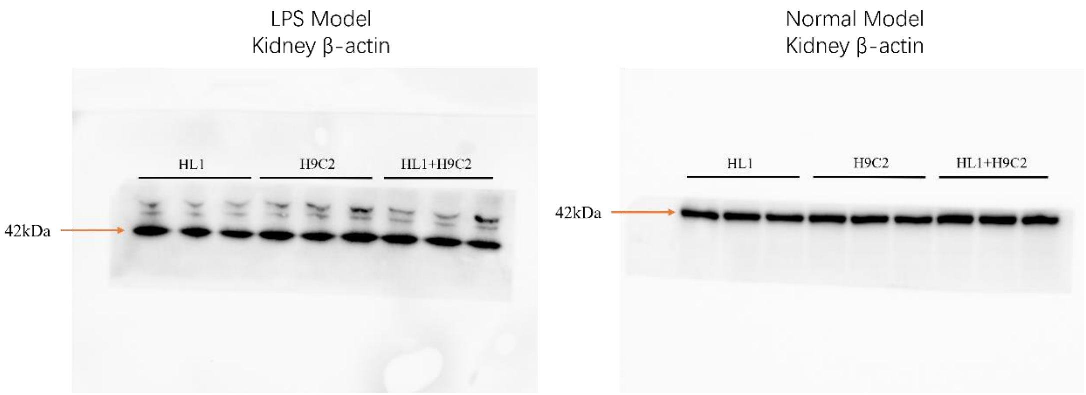

Figure 6I

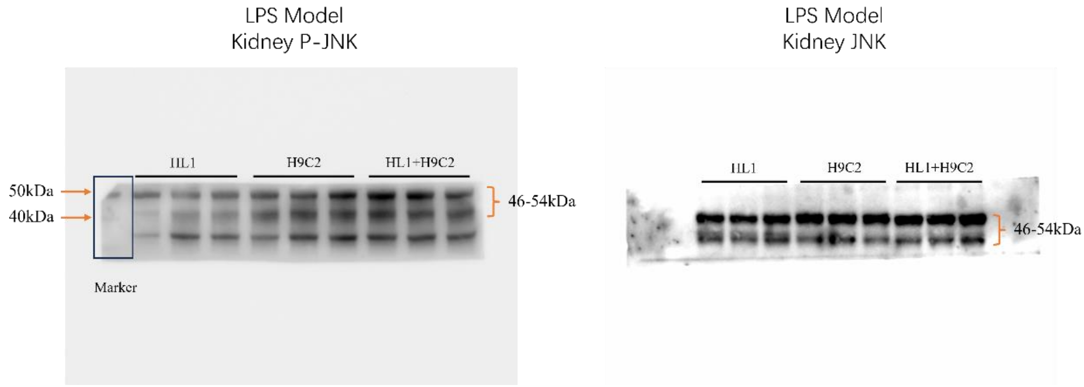

Figure 6I

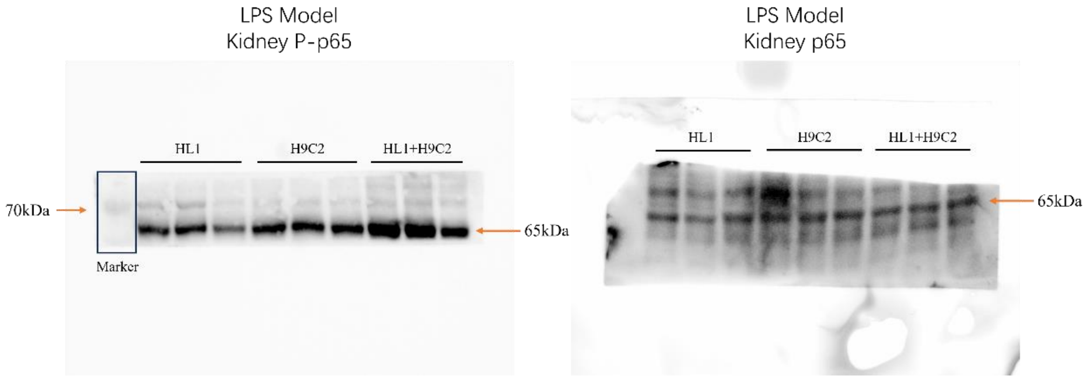

Supplement: Supplementary file 1 — WB raw data [file 41419_2025_7643_MOESM1_ESM.pdf]
