# Supplementary figures and images for: Mitochondrial transplantation: adaptive bio-enhancement
Source: Cell Death Dis. 2025 Jul 1;16(1):473. doi: 10.1038/s41419-025-07643-8 (PMC12218056; doi:10.1038/s41419-025-07643-8)

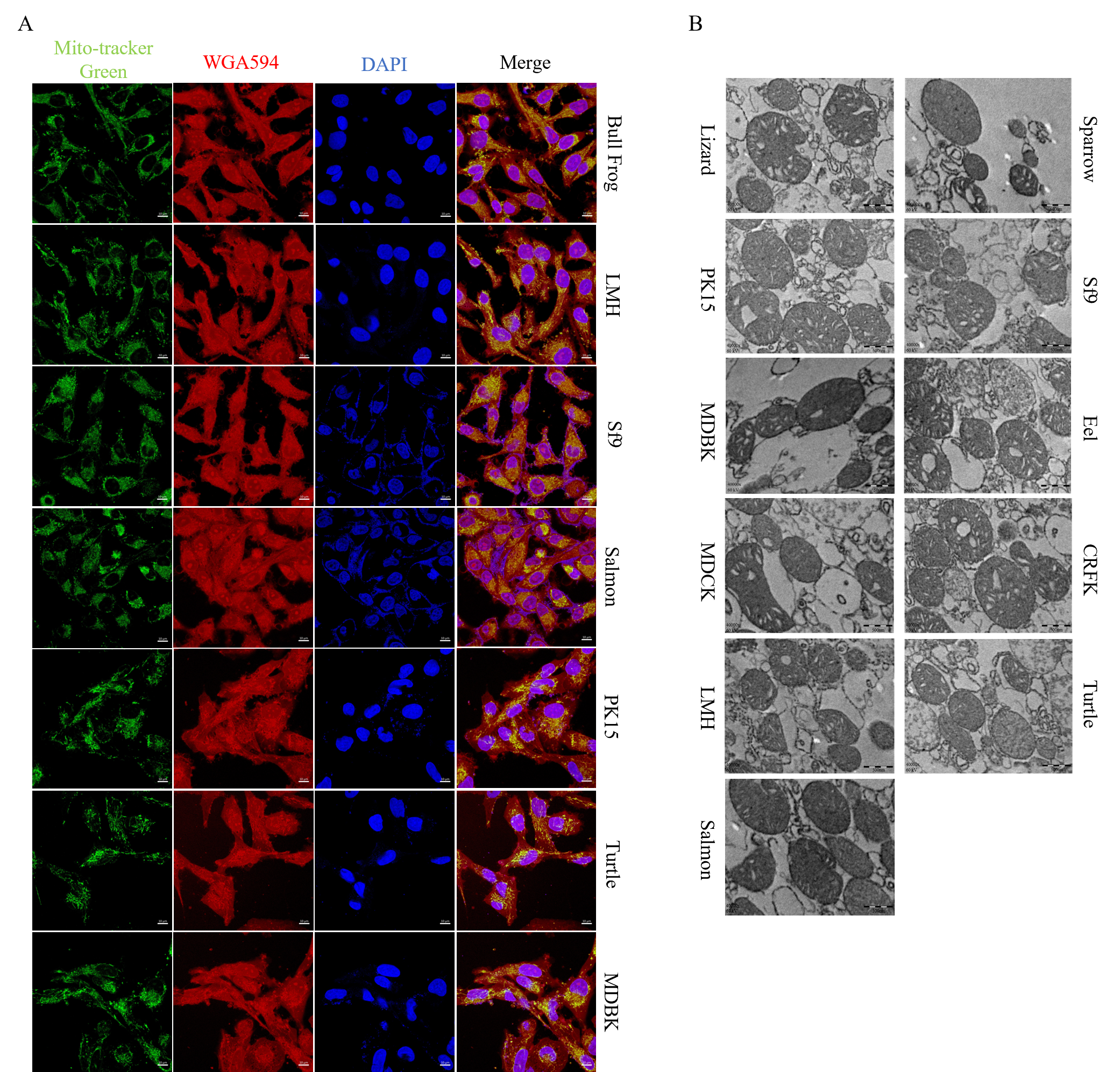

Supplement: Supplementary file 2 — Figure S1 [file 41419_2025_7643_MOESM2_ESM.tif]

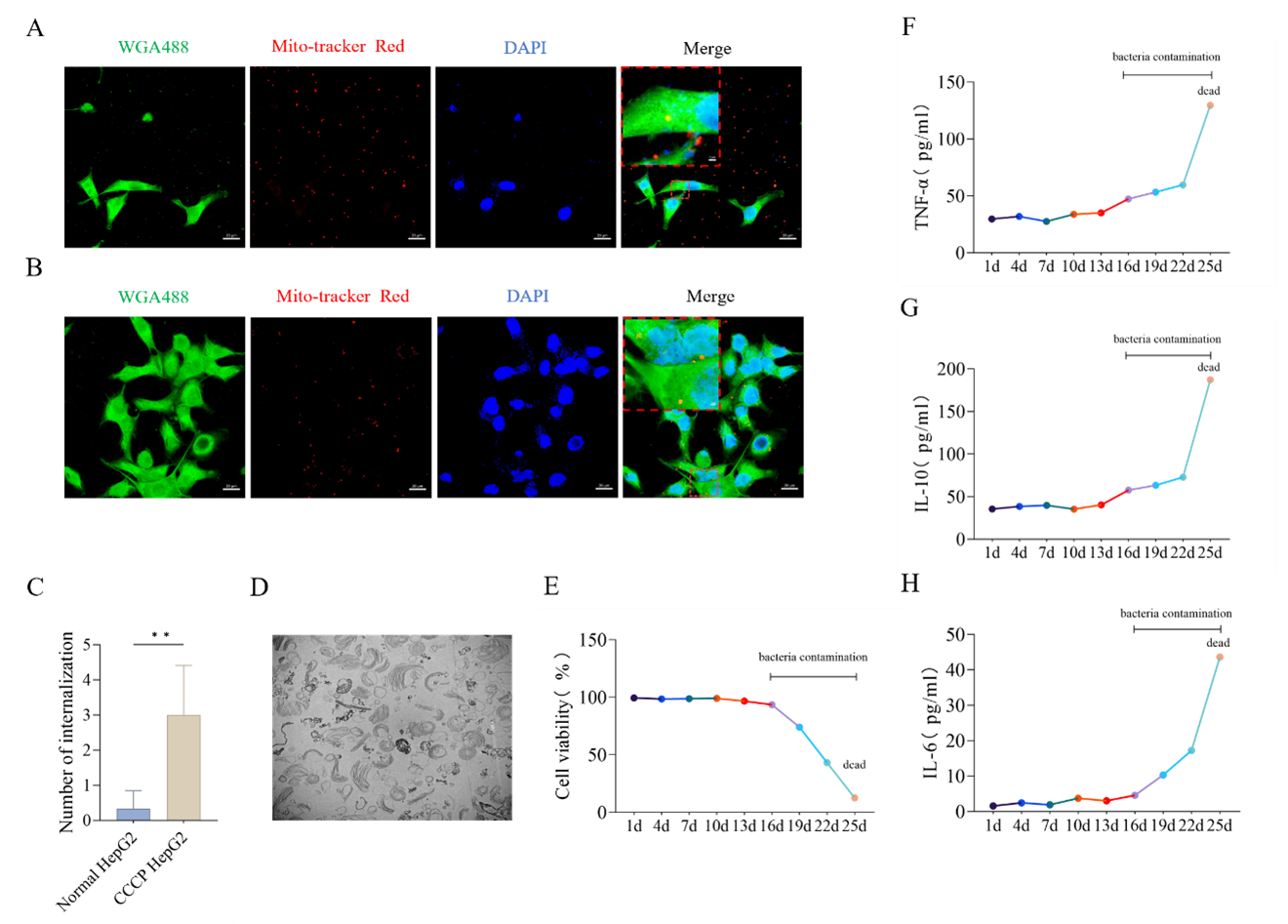

Supplement: Supplementary file 3 — Figure S2 [file 41419_2025_7643_MOESM3_ESM.tif]

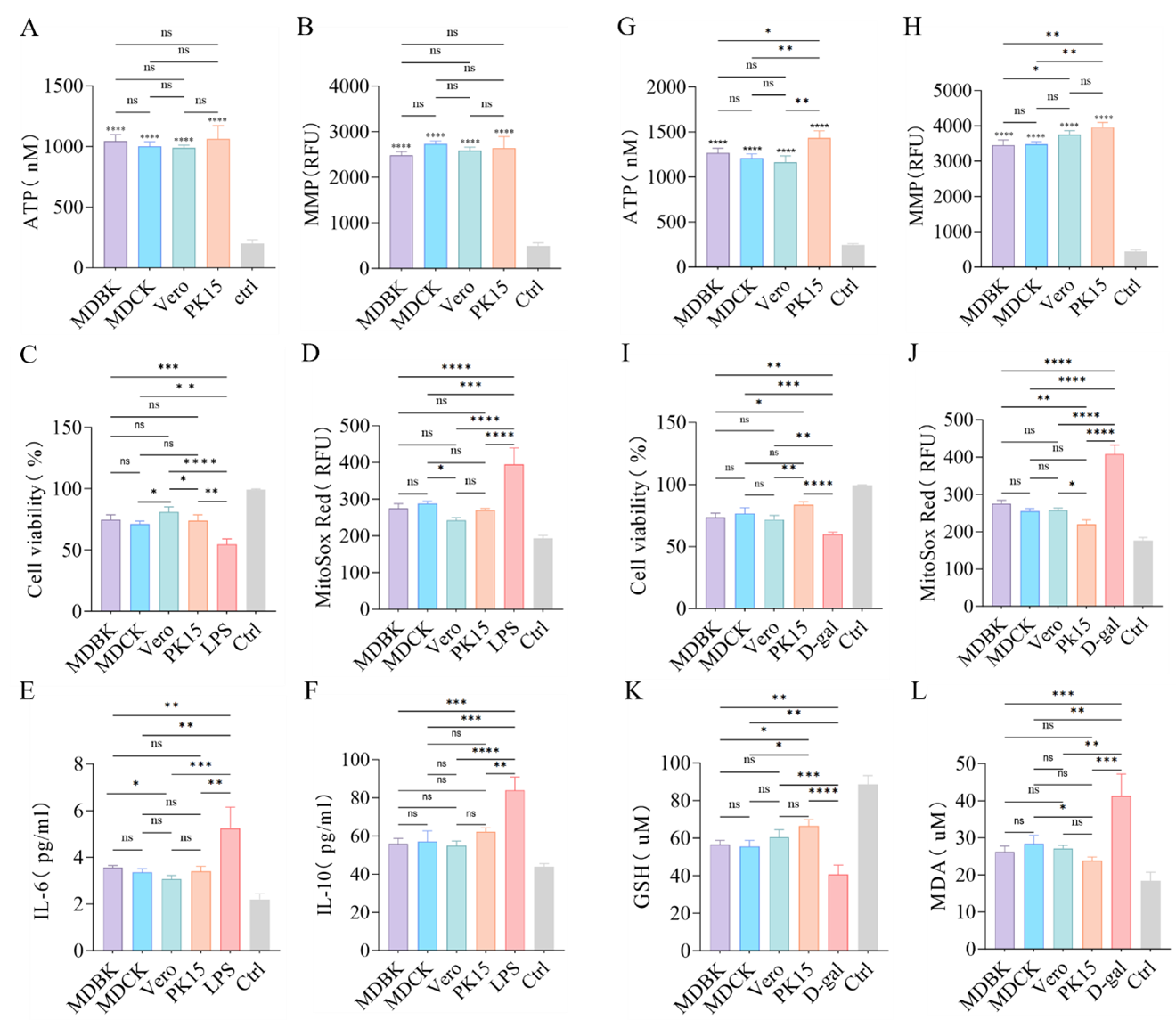

Supplement: Supplementary file 4 — Figure S3 [file 41419_2025_7643_MOESM4_ESM.tif]

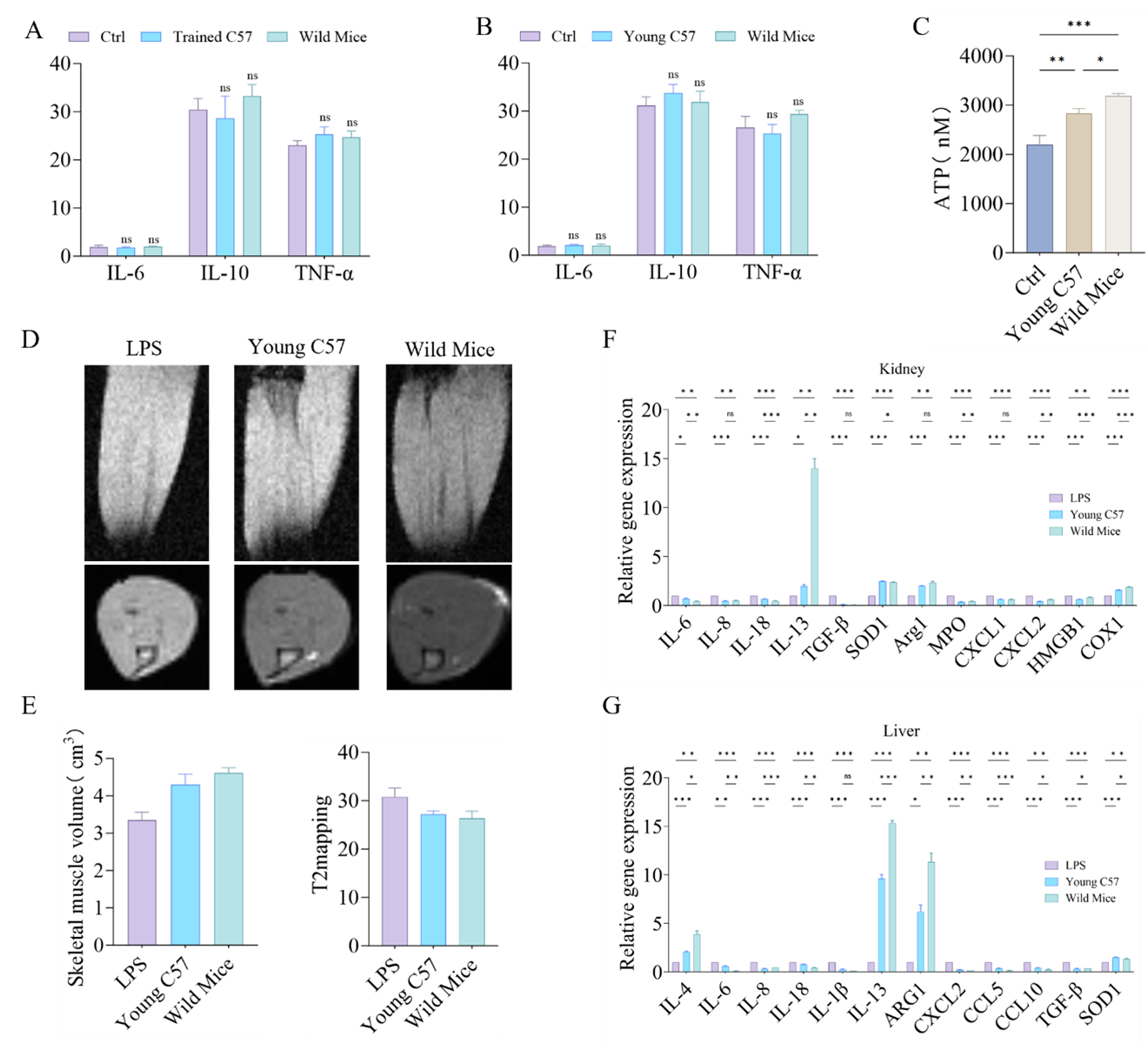

Supplement: Supplementary file 5 — Figure S4 [file 41419_2025_7643_MOESM5_ESM.tif]

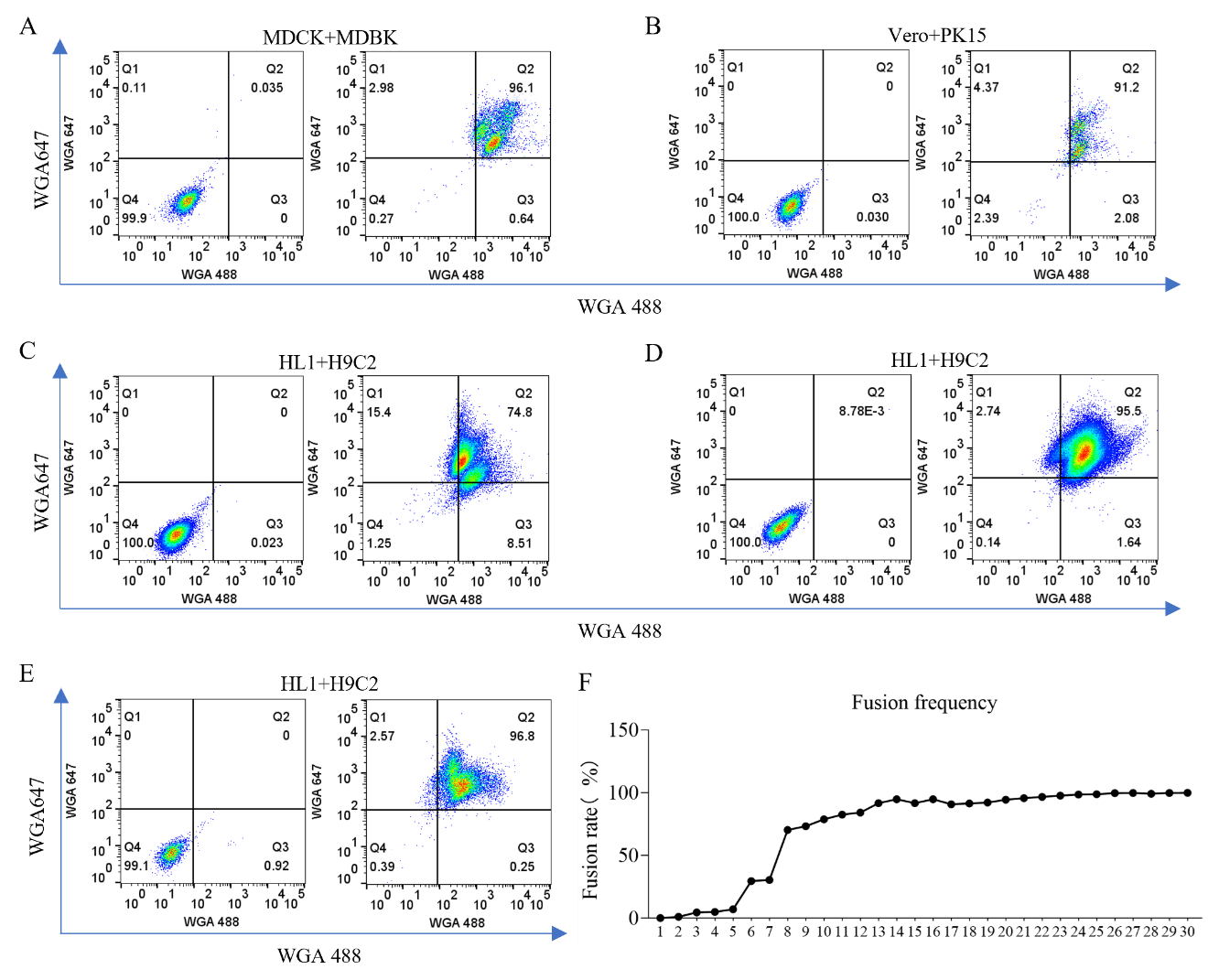

Supplement: Supplementary file 6 — Extend Figure 1 [file 41419_2025_7643_MOESM6_ESM.tif]

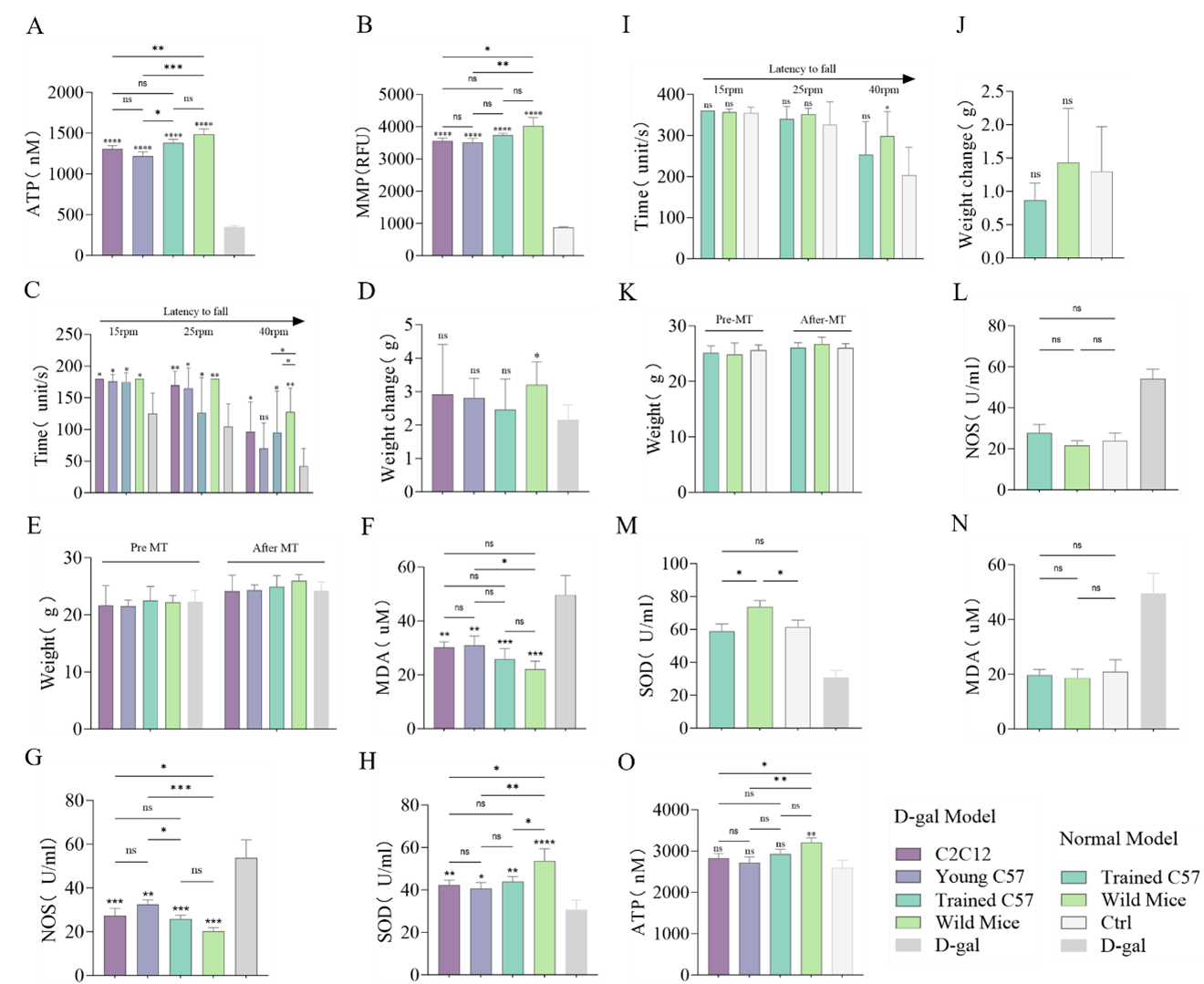

Supplement: Supplementary file 7 — Extend Figure 2 [file 41419_2025_7643_MOESM7_ESM.tif]

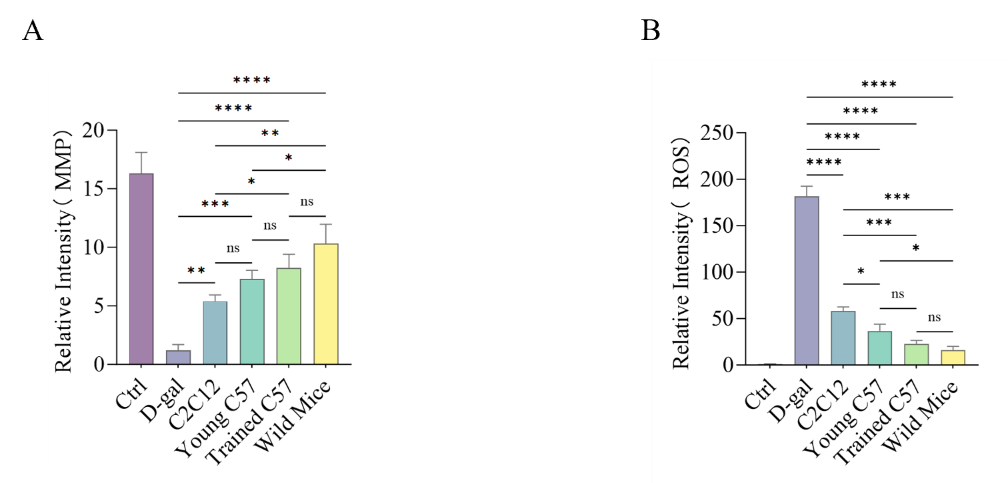

Supplement: Supplementary file 8 — Extend Figure 3 [file 41419_2025_7643_MOESM8_ESM.tif]
