# Supplementary material for: Mitochondrial transplantation: adaptive bio-enhancement
Source: Cell Death Dis. 2025 Jul 1;16(1):473. doi: 10.1038/s41419-025-07643-8 (PMC12218056; doi:10.1038/s41419-025-07643-8)
Supplement: Supplementary file 9 — Supplementary Material [file 41419_2025_7643_MOESM9_ESM.docx]

**Figure S1**

**Figure S1. Data related to Figure 1**

(A) Mitochondria labeled with green fluorescence using MitoTracker Green were transplanted from Salmon, Sf9, PK15, LMH, Turtle, MDBK and Bull Frog, and their colocalization with red fluorescence-labeled AC16, L929, and HepG2 cells stained with WGA594 was observed using fluorescence imaging after 24 hours. Blue fluorescence (DAPI) represents the cell nuclei. Scale bar: 10 µm. (B) Mitochondria from cells (MDBK, MDCK, CRFK, PK15, Sf9, LMH) and animals (Lizard, Sparrow, Salmon, Eel, Turtle) for electron microscopy to observe their morphology. Scale bar: 500 nm.

**Figure S2**

**Figure S2. Data related to Figure 1 and Figure 4**

(A) Co-localization fluorescence imaging of CCCP-treated HepG2 cells labeled with WGA488 and plant mitochondria labeled with Mito-Tracker Red, scale bar: 20 µm, magnified region scale bar: 2 μm. (B) Co-localization fluorescence imaging of normal HepG2 cells labeled with WGA488 and plant mitochondria labeled with Mito-Tracker Red, scale bar: 20 µm, magnified region scale bar: 2 μm. (C) Statistics of the number of Vaucheria litorea mitochondria internalized by normal HepG2 cells and CCCP-treated HepG2 cells, n=6. (D) Morphology of plant mitochondria observed under TEM, scale bar: 1 µm. (E) Changes in HepG2 cell viability after nine consecutive transplants of plant mitochondria, with untreated groups as control. (F-H) Changes in immune-inflammatory factors IL-6, IL-10, and TNF-α in HepG2 cells after nine consecutive transplants of plant mitochondria, with untreated groups as control.

**: p<0.01.

**Figure S3**

**Figure S3. Data related to Figure 2 and Figure 4**

(A, G) Assessment of ATP production capacity of isolated mitochondria, with mitochondria subjected to two freeze-thaw cycles at -80°C and 37°C to completely disrupt structure and function as control, n=3. (B, H) Analysis of mitochondrial membrane potential of isolated mitochondria, with mitochondria subjected to two freeze-thaw cycles at -80°C and 37°C to completely disrupt structure and function as control, n=3. (C) Changes in HepG2 cell viability after LPS treatment following transplantation of four different mitochondria, with untreated groups as control, n=3. (D) Effects of transplantation of four different mitochondria on mitochondrial ROS levels in LPS-treated HepG2 cells, with untreated groups as control, n=3. (E-F) Changes in IL-6 and IL-10 levels in HepG2 cells treated with LPS after transplantation of four different mitochondria, with untreated groups as control, n=3. (I) Changes in HepG2 cell viability after D-gal treatment following transplantation of four different mitochondria, with untreated groups as control, n=3. (J) Effects of transplantation of four different mitochondria on mitochondrial ROS levels in D-gal treated HepG2 cells, with untreated groups as control, n=3. (K-L) Changes in MDA and GSH levels in HepG2 cells treated with D-gal after transplantation of four different mitochondria, with untreated groups as control, n=3.

ns: no statistical significance, *: p<0.05, **: p<0.01, ***: p<0.001, ****: p<0.0001.

I extracted mitochondria from four types of cells and co-cultured them with LPS-treated HepG2 cells. When the overall mitochondrial functions were comparable (Figures S3A-B), the Vero group exhibited superior therapeutic effects. The Vero mitochondria significantly improved cell viability compared to the MDCK and PK15 groups (Figure S3C), reduced mitochondrial ROS production more effectively than the MDCK group (Figure S3D), and decreased IL-6 levels more substantially than the MDBK group (Figure S3E). Although there were no significant differences in IL-10 levels among the groups, the Vero group still demonstrated a slightly stronger effect (Figure S3F). These results indicate that Vero mitochondria provide better therapeutic outcomes for LPS-treated HepG2 cells, highlighting a more suitable and efficient match under these conditions.

I cultured P5 generation PK15, P15 generation MDBK, P15 generation MDCK, and P15 generation Vero cells to create mitochondria with varying bioenergetic strengths, which were then co-cultured with D-gal-treated HepG2 cells. Assessments of ATP and MMP assessments revealed that mitochondria from early-passage PK15 cells exhibited stronger bioenergetic function compared to other three groups (Figures S3G-H). PK15 mitochondria significantly enhanced HepG2 cell viability (Figure S3I) and reduced ROS production (Figure S3J) in comparison to MDBK and Vero groups. Additionally, they increased GSH levels than MDCK and MDBK groups (Figure S3K), and decreased MDA production more effectively than MDCK mitochondria (Figure S3L), thereby reducing oxidative stress in HepG2 cells. This further confirms the therapeutic advantage of mitochondria with more powerful bioenergetic function.

**Figure S4**

**Figure S4. Data related to Figure 5 and Figure 6**

(A) Analysis of immune-inflammatory factors IL-6, IL-10, and TNF-α in normal mice 24 hours after mitochondrial transplantation from the first animal experiment, n=3. (B) Analysis of immune-inflammatory factors IL-6, IL-10, and TNF-α in normal mice 24 hours after mitochondrial transplantation from the second animal experiment, n=3. (C) Changes in normal BMDM ATP productivity after transplantation of two different mitochondria in the second animal experiment, with untreated groups as control, n=3. (D-E) MRI assessment of hindlimb muscle volume and muscle damage repair in acute inflammation model mice one week after transplantation of different mitochondria in the second animal experiment, n=2. (F) Real-time PCR analysis of renal expression of IL-6, IL-8, IL-18, IL-13, TGF-β, SOD1, ARG1, MPO, CXCL1, CXCL2, HMGB1, and COX1 in acute inflammation model mice after transplantation of different mitochondria in the second animal experiment, n=5. (G) Real-time PCR analysis of hepatic expression of IL-4, IL-6, IL-8, IL-18, IL-1β, IL-13, TGF-β, SOD1, ARG1, CCL5, CCL10, CXCL2, and HMGB1 in acute inflammation model mice after transplantation of different mitochondria in the second animal experiment, n=5.

ns: no statistical significance, *: p<0.05, **: p<0.01, ***: p<0.001, ****: p<0.0001

**Extend Figure1**

**Extend Figure1**: **Statistical analysis of cell fusion**

(A) Fusion analysis of WGA647-labeled MDBK cells and WGA488-labeled MDCK cells using flow cytometry. (B) Fusion analysis of WGA647-labeled Vero cells and WGA488-labeled PK15 cells using flow cytometry. (C-E) Fusion analysis of WGA647-labeled HL1 cells and WGA488-labeled H9C2 cells using flow cytometry. (D) Changes in cell fusion rate with improvements in experimental techniques.

**Extend Figure2**

**Extend Figure2：Mitochondrial Transplantation Enhances Physical Function and Biological Potency in Mice**

(A-B) Measurement of ATP production capacity and membrane potential in isolated mitochondria. Ctrl: Mitochondria subjected to two freeze-thaw cycles at -80°C and 37°C, used for comparison, n=3. (C) Changes in motor ability of D-gal model mice following transplantation of four different types of mitochondria, n=8. (D-E) Changes in body weight of D-gal model mice following transplantation of four different types of mitochondria, n=8. (F-H) Evaluation of the antioxidant and oxidative capacity of D-gal model mice by measuring serum glutathione content, superoxide dismutase activity, and malondialdehyde levels after transplantation of four different types of mitochondria, n=3. (I) Changes in motor ability of normal model mice following transplantation of four different types of mitochondria, n=6. (J-K) Effects of transplantation of two different types of mitochondria on the motor ability of normal mice. Ctrl: Untreated group, n=6. (L-N) Evaluation of the antioxidant and oxidative capacity of normal mice by measuring serum GSH content, SOD activity, and MDA levels after transplantation of two different types of mitochondria. Ctrl: Untreated group, n=3. (O) Changes in ATP production capacity in normal HepG2 cells after transplantation of four different types of mitochondria. Ctrl: Untreated group, n=3.

ns = no statistical significance, * = p<0.05, ** = p<0.01, *** = p<0.001, **** = p<0.0001

In the first animal experiment, I utilized a D-gal-induced mouse aging model and intravenously injected mitochondria extracted from mouse myoblasts (C2C12), gastrocnemius tissues of three-month-old C57BL/6J mice, HIIT-trained C57BL/6J mice and wild mice into aged mice. At the same time, I injected mitochondria from HIIT-trained C57BL/6J and wild mice gastrocnemius tissues into five-month-old normal C57BL/6J mice. Additionally, mitochondria from each group were also co-cultured with normal HepG2 and D-gal-treated HepG2 cells. ATP and MMP assays were employed to evaluate mitochondrial function. The Wild Mice group exhibited significant differences compared to the C2C12 and Young C57 groups, while the Trained C57 group showed a significant difference from the Young C57 group. Although no significant difference was observed between the Wild Mice and Trained C57 groups, the overall performance of the Wild Mice group was slightly superior (Extend Figures 2A-B). No immune or inflammatory responses were detected 24 hours post-transplant, as indicated by measurements of IL-6, IL-10, and TNF-α (Figure S4A).

In the aging model, exercise performance improved across all groups three days after mitochondrial transplantation. The Wild Mice group exhibited the best long-term exercise endurance (Extend Figure 2C). In the normal model, only the Wild Mice group showed an increase in exercise capacity (Extend Figure 2I). One week post-transplantation, body weight significantly increased in the aging model, with the Wild Mice group achieving the most substantial recovery (Extend Figures 2D-E). In contrast, no significant weight differences were observed in the normal model (Extend Figures 2J-K).

Despite the beneficial effects observed across all transplantation groups, the Wild Mice group demonstrated a more pronounced reduction in oxidative stress among aging mice. The MDA levels in the Wild Mice group were significantly lower compared to those in the Young C57 group (Extend Figures 2F). NOS levels were also lower in the Wild Mice group than in the C2C12 and Young C57 groups. Although the Trained C57 group exhibited differences from the Young C57 group, it remained inferior to the Wild Mice group (Extend Figure 2G). SOD activity in the Wild Mice group was higher than in the C2C12, Young C57, and Trained C57 groups (Extend Figure 2H), and Wild Mice mitochondria significantly enhanced SOD activity in normal mice (Extend Figure 2M).

Furthermore, all mitochondrial transplantation groups improved bioenergetics in normal HepG2 cells, with the Wild Mice exhibiting the most significant increases (Extend Figure 2O). Four kinds of mitochondrial transplantation restored membrane potential and reduced ROS generation in D-gal-treated HepG2 cells, with the Wild Mice and Trained C57 groups demonstrating a superior ability to alleviate oxidative stress and restore mitochondrial membrane potential (Extend Figures 3A-B).

**Extend Figure3**

**Extend Figure3：****Mitochondrial Transplantation Enhances the Biological Function of HepG2 Cells**

(A)The JC-1 statistical analysis method was employed to assess the recovery of mitochondrial membrane potential fluorescence intensity in HepG2 cells treated with D-gal after four different species of mitochondrial transplantation. Untreated cells served as controls, n = 3. (B) Statistical analysis was conducted to evaluate the effects of mitochondrial transplantation from four different species on the fluorescence intensity of reactive oxygen species (ROS) in D-gal-treated HepG2 cells, with untreated cells again serving as controls, n = 3.

ns = no statistical significance, * = p<0.05, ** = p<0.01, *** = p<0.001, **** = p<0.0001
